# The role of selenium, vitamin C, and zinc in benign thyroid diseases and of selenium in malignant thyroid diseases: Low selenium levels are found in subacute and silent thyroiditis and in papillary and follicular carcinoma

**DOI:** 10.1186/1472-6823-8-2

**Published:** 2008-01-25

**Authors:** Roy Moncayo, Alexander Kroiss, Manfred Oberwinkler, Fatih Karakolcu, Matthias Starzinger, Klaus Kapelari, Heribert Talasz, Helga Moncayo

**Affiliations:** 1 WOMED, Karl-Kapferer-Strasse 5, Innsbruck, Austria; 2 Clinical Department of Nuclear Medicine, Medical University of Innsbruck, Austria; 3 Clinical Department of Pediatrics, Medical University of Innsbruck, Austria; 4 Biocenter, Division of Clinical Biochemistry, Innsbruck Medical University, Austria

## Abstract

**Background:**

Thyroid physiology is closely related to oxidative changes. The aim of this controlled study was to evaluate the levels of nutritional anti-oxidants such as vitamin C, zinc (Zn) and selenium (Se), and to investigate any association of them with parameters of thyroid function and pathology including benign and malignant thyroid diseases.

**Methods:**

This controlled evaluation of Se included a total of 1401 subjects (1186 adults and 215 children) distributed as follows: control group (n = 687), benign thyroid disease (85 children and 465 adults); malignant thyroid disease (2 children and 79 adults). Clinical evaluation of patients with benign thyroid disease included sonography, scintigraphy, as well as the determination of fT3, fT4, TSH, thyroid antibodies levels, Se, Zn, and vitamin C. Besides the routine oncological parameters (TG, TSH, fT4, ultrasound) Se was also determined in the cases of malignant disease. The local control groups for the evaluation of Se levels were taken from a general practice (WOMED) as well as from healthy active athletes. Blood samples were collected between 8:00 and 10:30 a.m. All patients lived in Innsbruck. Statistical analysis was done using SPSS 14.0. The H_o _stated that there should be no differences in the levels of antioxidants between controls and thyroid disease patients.

**Results:**

Among the thyroid disease patients neither vitamin C, nor Zn nor Se correlated with any of the following parameters: age, sex, BMI, body weight, thyroid scintigraphy, ultrasound pattern, thyroid function, or thyroid antibodies. The proportion of patients with benign thyroid diseases having analyte concentrations below external reference cut off levels were 8.7% of cases for vitamin C; 7.8% for Zn, and 20.3% for Se. Low Se levels in the control group were found in 12%. Se levels were significantly decreased in cases of sub-acute and silent thyroiditis (66.4 ± 23.1 μg/l and 59.3 ± 20.1 μg/l, respectively) as well as in follicular and papillary thyroid carcinoma. The mean Se level in the control group was 90.5 ± 20.8 μg/l.

**Conclusion:**

The H_0 _can be accepted for vitamin C and zinc levels whereas it has to be rejected for Se. Patients with benign or malignant thyroid diseases can present low Se levels as compared to controls. Low levels of vitamin C were found in all subgroups of patients.

## Background

During thyroid hormone synthesis, thyroid tissue is exposed to H_2_O_2 _making it imperative that protective systems can prevent damage to the gland [1-5]. This tissue protection can be achieved by selenium-dependent products, e.g. the glutathione peroxidase [6-8]. Serum levels of selenium (Se) are considered to depict the adequacy regarding GPx levels and activity [9-11]. In addition to this, the determination of selenoproteins, e.g. selenoprotein P (SePP), can deliver further information on the adequacy of Se levels [12].

Several investigators have previously evaluated Se levels in patients with benign and malignant thyroid diseases. These studies demonstrated that hyperthyroidism is associated with decreased levels of Se [13-15]. Derumeaux et al. [16] also concluded that Se might have a protective role in relation to goiter, and that thyroid hypogenicity appears to be related to Se levels [16]. In the field of malignant thyroid diseases, decreased tissue levels of zinc (Zn) and Se, appear to be associated with carcinoma [17]. Studies based on the Janus serum bank have shown the value of pre-diagnostic levels of Se in relation to thyroid carcinoma [18,19].

In experimental settings other micronutrients such as Zn have also been shown to play a role on thyroid morphology and metabolism [20]. It should be kept in mind that Zn is also an antioxidant [21], while at the same time it has important functions in relation to immune function [22,23]. Comparable functions have been described for Zn and Se together [24,25]. In addition to this, Martin et al. have described a positive interaction between vitamin C and Se homeostasis [26].

Besides these thyroid-related data one has to consider the importance of nutrient antioxidants in a much wider frame, i.e. in relation to their protective effects on health [27-30]. An example of this approach is the French SU.VI.MAX study, where micronutrients have been evaluated in relation to cardiovascular diseases, thyroid disease, and cancer [31,32]. One central thought behind these studies has been stated as follows: "Insufficient intake of antioxidant vitamins and minerals may reduce the body's capacity to defend itself from the effect of activated oxygen derivatives on cell processes that play a role in the development of cancer and cardiovascular disease [33,34]." It follows that there is a clinical relevance in the determination of base line levels of nutrient antioxidants. For this reason, we designed this investigation in order to evaluate the levels of nutritional anti-oxidants in a controlled study, i.e. comparing patients with controls.

## Methods

### Laboratory determinations of nutritional antioxidants

Blood samples were collected between 8:00 and 10:30 a.m. After centrifugation (1500 g, 15 minutes) serum was removed and aliquots were frozen and stored at -20°C. Selenium concentrations in human serum were determined using a graphite furnace atomic absorption spectrometer (Unicam Model Solaar, 939 QZ, Cambridge, UK) with Zeeman background correction and pyrolytic carbon-coated graphite tubes. Slit 0.5 and wavelength λ 196.0 nm were used as spectrometer parameters. Specimens were diluted 1:10 with 0.1% HNO3, 0.05% Triton ×100, and 0.05% silicone anti-foaming agent (Merck, Germany). Palladium nitrate as matrix modifier and matrix-matched standards for calibration (6.25, 12.5, 25, and 50 μg/l) were used. Multiple aliquots of a control pooled serum sample were analyzed during each batch of analyses (intra-assay CV < 4.5%). Imprecision is shown as coefficient of variation (CV). In addition, an external control, (ClinChek-Serum control, Lot No. 323; Recipe Chemicals and Instruments, Munich, Germany), was analyzed with each batch. Analyzed values were within the expected range given by the manufacturer (i.e., Level1: 46–78 μg/L, certified mean concentration: 62.0 μg/L; Level 2: 84–140 μg/L, certified mean concentration: 112 μg/L), with means of 63.8 ± 6.15 μg/L (n = 19, CV 9.6%) for Level 1, and 109.9 ± 10.15 μg/L (n = 20, CV 9.2%) for Level 2 serum analyses.

Serum zinc content was investigated according to Smith et al. [35] by employing a flame (air-acetylene burner) atomic absorption spectrometer (Unicam Model Solaar, 939, Cambridge, UK). Bandpass 0.5 nm and wavelength λ 213.9 nm were used as spectrometer parameters. Specimens were diluted 1:10 or 1:5 with double distilled water. Working standards of zinc containing 0.1, 0.25, 0.5, and 1.0 μg/ml were prepared from stock standard solution containing 1000 μg Zn/ml. Multiple aliquots of a control pooled serum sample were analyzed during each batch of analyses (intra-assay CV < 3.5%). In addition, an external control, (ClinChek-Serum control, Lot No. 323; Recipe Chemicals and Instruments, Munich, Germany) was analyzed with each batch. Analyzed values were within the expected range given by the manufacturer (i.e., Level 1: 636–1060 μg/L, certified mean concentration: 848 μg/L; Level 2: 1706–2844 μg/L, certified mean concentration: 2275 μg/L), with means of 798 ± 79 μg/L (n = 20, CV 9.9%) for Level 1, and 2037 ± 135 μg/L (n = 20, CV 6.7%) for Level 2 serum analyses.

For the determination of ascorbic acid a 100 μl volume of serum was stabilized immediately after preparation by addition of 100 μl 10% metaphosphoric acid and incubated for 10 minutes at 4°C. Thereafter the sample was centrifuged at 10000 × g and 4°C. 20 μl of the supernatant was used for determination of ascorbic acid. The HPLC method used (flow rate 1.0 ml/min, detection at 245 nm) is based on the work published by Esteve et al. [36] with some modifications. The ascorbic acid standard solution (10 mg/l) was prepared daily in deionized water Millipore-Milli Q. 4-hydroxyacetanilide was used as internal standard. Multiple aliquots of a control pooled serum sample were analyzed (intra-assay CV < 3.0%). In addition, an external quality control (Chromsystems Vitamin C Plasma control, Lot No. 1906; Chromsystems Instruments and Chemicals GmbH, Munich, Germany), was analyzed with each batch (inter-assay CV < 5.0%). Determinations of selenium, zinc and ascorbic acid were made in duplicate, and the analysts were blinded as to the case status of the specimens.

### Vitamin C and zinc in patients with benign thyroid disease

In an initial phase a group of 425 consecutive adult patients attending the out-patient unit of the Clinical Department of Nuclear Medicine of the Medical University of Innsbruck were investigated. In these patients the levels of Zn and vitamin C (n = 415) were determined. The research protocol was approved by the Institutional Ethics Committee. Informed consent was obtained from each subject. Clinical evaluation included sonography, scintigraphy, and thyroid function parameters. Determinations of fT3, fT4, TSH and thyroid antibodies levels were done on the Advia Centaur system (Bayer Health Care, Austria) as described elsewhere [37].

Weight, height and medication were recorded for each patient. Thyroid ultrasound was done using a Siemens Sonoline Antares equipped with a VFX13-5 transducer (Siemens, Erlangen, Germany). RM carried out the ultrasound examinations. Thyroid scintigraphy was done on a Siemens Orbiter gamma camera (Siemens, Erlangen, Germany) after i.v. application of 37 MBq of ^99m^Tc pertechnetate. Based on the clinical history, the laboratory tests and the imaging procedures, a final diagnosis was given in each case. The final diagnoses included: normal thyroid function (negative thyroid serology), Graves' disease (positive TSH receptor antibodies), Hashimoto's disease (positive thyroid serology), sub-acute thyroiditis and silent thyroiditis [38]. Thyroid function was classified as normal, hypothyroid or hyperthyroid based on the measured levels of fT3, fT4 and TSH [37,39,40]

### Se levels in patients with thyroid disease

The study included 465 adult patients and 85 pediatric patients presenting benign thyroid disease, and 164 patients with malignant thyroid disease taking thyroid medication. Laboratory and ultrasound examinations were the same as described above. Thyroid scintigraphy was done neither in the pediatric group nor in the group with thyroid carcinoma.

### Control groups for the evaluation of Se

A control group for the evaluation of Se levels was taken from a general practice (WOMED, n = 554; 468 females, mean age 41 ± 11 years; 46 males, mean age 37 ± 15 years). These patients were clinically investigated by RM and HM. The geographical origin of this control group was similar to that of the thyroid patients, i.e. all subjects lived in the province of Tirol in Austria. By this, differences due to different geographical origin were minimized. In this group of patients the following laboratory investigations were available: kidney function tests, liver function tests, red and white cell counts, electrolytes, and thyroid function tests. The major diagnoses in this group were: burn-out syndrome, depression, infertility, menopause, GI disorder and DM. None of these entities is known to be associated with alterations in Se levels. A second group of medically controlled subjects included 133 active winter sport athletes not having overt thyroid disease. This group included 44 females and 89 males with a mean age of 18.7 ± 4.5 years. Regular medical examinations did not reveal any pathology that can be associated to changes in Se levels. In the subgroup of 24 ski jumpers, a total of additional 124 biochemical parameters including red and white blood counts, liver function, kidney function, electrolytes, immunoglobulins, iron metabolism, vitamin B as well as an aminogram were analyzed. Correlation studies were done in relation to Se levels.

### Statistical analysis

Statistical analysis was done using SPSS version 14. Explorative analyses of correlation were carried out between diagnostic parameters of thyroid function (fT3, fT4, TSH, and thyroid antibodies) and the measured levels of antioxidants. Thyroid characteristics as seen in ultrasound and scintigraphy were coded and compared by cross tabulation with the categorized levels of nutritional antioxidants. In order to allow for qualitative comparison with recent data dealing with the nutritional status of adult Europeans, the cut-off levels for Se, Zn, and vitamin C were taken from Galan et al. [34]. These levels correspond to: 0.75 μM for Se; 10.7 μM for Zn; and 11.4 μM for vitamin C. Additional external reference levels for Se were taken from publications that have evaluated the concentration of Se needed to achieve full expression of Se related products, i.e. deiodinase, glutathione peroxidase (GPx) and selenoprotein P (SePP) [41-43]. Results were categorized as being below or above these cut off levels as well as a percentual fraction of the reference levels. Finally, differences among the diagnostic groups for each parameter were analyzed by one way ANOVA using Bonferroni's correction. For all evaluations a p-value < 0.01 was taken as significant. The H_o _stated that there are no differences in the levels of antioxidants between controls and thyroid disease patients.

## Results

### General aspects

Neither vitamin C, nor Zn, nor Se levels showed any correlation with sex, age, weight, height, BMI, thyroid function, ultrasound pattern, nor thyroid morphology. Lack of association between Se and BMI was also seen even after classifying the BMI results into subgroups as has been done recently by Meplan et al. [44]. The descriptive statistics of BMI levels in our study were: mean 23.57 ± 4.5 kg/m^2 ^(S.D.), the 95^th ^percentile corresponded to a value of 31.7 kg/m^2^.

In the group of patients with benign thyroid diseases there were no differences in the levels of Zn or vitamin C according to the classification of disease type (Table 1). Due to this lack of correlation, determinations of vitamin C and Zn were not carried out in the subsequent cases, i.e. pediatric cases and in the group of patients with thyroid malignancy.

**Table 1:** Zn and vitamin C levels in the patients with benign thyroid disease

		**n**	**mean**	**S.D.**	**SEM**	**95% confidence interval**		**minimum**	**maximum**
		
						**low**	**high**		
**Zinc**	**Normal thyroid**	276	0,95	0,20	0,01	0,92	0,97	0,41	1,66
	**Immunogenic thyroid disease**	118	0,97	0,19	0,02	0,94	1,01	0,60	1,43
	**Subacute thyroiditis**	31	0,96	0,21	0,04	0,88	1,04	0,42	1,48
	**All**	425	0,96	0,20	0,01	0,94	0,98	0,41	1,66
**Vit. C**	**Normal thyroid**	273	6,07	2,94	0,18	5,72	6,42	1,80	13,6
	**Immunogenic thyroid disease**	112	6,10	2,63	0,25	5,61	6,59	1,10	13,2
	**Subacute thyroiditis**	29	6,48	3,12	0,58	5,29	7,66	0,70	12,8
	**All**	414	6,11	2,87	0,14	5,83	6,39	0,70	13,6

### Comparison of analyte levels with published threshold values – the nutritional aspect of the study

In spite of the lack of association of between vitamin C or Zn levels in relation to thyroid disease it should be stressed that the overall proportion of thyroid patients having vitamin C concentrations below the threshold levels reported in the SU.Vi.MAX study [34] was 8.4% of cases for vitamin C and 7.73% for Zn. The figure for Zn is similar to the reported one, the figure for vitamin C exceeds the levels reported.

The percentage of subjects having low levels of Se as compared to the functional threshold level known to be necessary for the maintenance of optimal plasma glutathione peroxidase [45] is shown in Table 2. In this qualitative comparison the largest proportions of cases having low Se levels were seen in children with hyperthyroidism and polyendocrinopathy, as well as in adults with subacute and silent thyroiditis, and in follicular carcinoma.

**Table 2:** Percentage of subjects with Se levels below the threshold for optimal plasma glutathione peroxidase [45]

	**% of patients with**	
	
**Study groups**	**low Se**	**sufficient Se**
Controls	33,0	67,0
Ski jumpers	50,0	50,0
Ski runners	43,1	56,9
Children hypothyroidism	48,9	51,1
Children Hashimoto	46,4	53,6
Children hyperthroidism	75,0	25,0
Children polyendocrinopathy	66,7	33,3
Thyroid normal	49,5	50,5
Thyroid immunogenic	46,6	53,4
Subacute thyroiditis	76,0	24,0
Silent thyroiditis	84,6	15,4
follicular Ca	64,3	35,7
foll papillary Ca	63,6	36,4
giant cell papillary Ca	40,0	60,0
invasive Ca	75,0	25,0
papillary Ca	52,1	47,9

Table 3 shows another qualitative analysis in relation to the threshold levels known to be necessary for optimal deiodinase activity, plasma glutathione peroxidase and selenoprotein P levels [41,42]. While the mean Se value found in our study was sufficient to cover the requirements for deiodinase activity, it was not sufficient neither for platelet glutathione peroxidase nor for selenoprotein P expression.

**Table 3:** Se levels expressed in percentage of threshold levels in relation to deiodinases (IDI), plasma glutathione peroxidase (GPx), and selenoprotein P (SePP) [41,42]

	**measured mean Se**	**Se related parameters and their threshold levels in μM**		
	
		**IDI**	**GPx**	**SePP**
	
	**μM**	0,82	1,2	1,7
Controls	1,14	139,51 *	95,33	67,29
Ski jumpers	1,05	128,21	87,61	61,84
Ski runners	1,09	133,50	91,22	64,39
Children Hypothyroidism	1,04	126,38	86,36	60,96
Children Hashimoto	0,98	119,33	81,54	57,56
Children hyperthyroidism	0,83	101,67	69,48	49,04
Children Poly	0,85	103,19	70,51	49,77
Thyroid normal	1,02	124,97	85,40	60,28
Thyroid immunogenic	1,04	126,39	86,37	60,96
Subacute thyroiditis	0,84	102,29	69,90	49,34
Silent thyroiditis	0,75	91,40	62,45	44,09
anaplastic Ca	1,20	146,46	100,08	70,64
follicular Ca	0,97	118,54	81,00	57,18
foll.-papillary Ca	0,87	106,14	72,53	51,20
giant cell papillary Ca	1,06	129,02	88,16	62,23
invasive Ca	0,95	115,31	78,79	55,62
microcarcinoma	0,86	104,98	71,74	50,64
papillary Ca	1,02	123,95	84,70	59,79
unclassified Ca	0,96	117,02	79,96	56,45
All (n = 1401)	1,06	129,81	88,71	62,62

*: the figures are given as percent of the threshold level (% = measured Se concentration * 100/threshold level)

### Quantitative analyses of the Se status

#### Control group

Initial one-way ANOVA showed no difference in the Se levels in relation to the diagnostic subgroups contained in the adult control group (burn-out, depression, infertility, menopause, GI disorders, and DM). It should be mentioned that none of these conditions is known to produce alterations in the concentration of Se. The mean Se level for the non-thyroid disease control group was 90.5 ± 20.8 μg/l; the 5^th ^percentile corresponded to 65.7 μg/l, and the 95^th ^percentile to 129 μg/l. The control group consisting of active athletes did not differ from the adult control group.

### Selenium status in benign and malignant thyroid disease

One-way analysis of variance of all Se samples, i.e. thyroid vs. non-thyroid disease patients, revealed significantly diminished levels (p < 0.001) for all thyroid disease patients. Among the groups of patients with benign thyroid disease, the Se levels of those with subacute and with silent thyroiditis were significantly lower as compared to the other diagnostic groups. In sub-acute and silent thyroiditis the measured levels were: 66.4 ± 23.1 μg/l and 59.3 ± 20.1 μg/l, respectively. In the groups of patients with thyroid malignancy, those presenting follicular and papillary types of carcinoma showed also diminished Se concentrations (76.9 ± 21.1 μg/l and 80.4 ± 19.8 μg/l, respectively). Children with thyroid disease and polyendocrinopathy had low Se levels similar to those found in adults with thyroiditis (mean 66.9 μg/l). The minimal levels of Se were observed in the group of adult patients with benign thyroid disease (range 20.7 to 30 μg/l) (Table 4).

**Table 4:** One-way ANOVA (Bonferroni) analysis of the Se levels

	**n**	**mean**	**S.D.**	**SEM**	**95% confidence interval**		**minimum**	**maximum**	**p-value vs controls**
		
					**low**	**high**			
Controls	554	90,49	20,85	0,89	88,75	92,23	47,34	187,20	
Ski jumpers	24	83,17	26,51	5,41	71,97	94,36	51,30	136,00	1
Ski runners	109	86,59	21,04	2,02	82,60	90,59	45,82	165,00	1
Children hypothyroidism	47	81,98	22,53	3,29	75,36	88,59	41,30	131,00	1
Children Hashimoto	28	77,40	23,54	4,45	68,27	86,53	40,50	119,30	0,308
Children hyperthyroidism	4	65,95	13,99	6,99	43,69	88,21	53,40	81,00	1
Children polyendocrinopathy	6	66,93	20,92	8,54	44,98	88,89	43,70	96,20	1
Thyroid normal adults	283	81,06	21,62	1,29	78,53	83,59	30,10	172,80	< 0,001
Thyroid immunogenic adults	131	81,98	24,82	2,17	77,69	86,27	28,50	163,00	0,009
Subacute thyroiditis adults	25	66,35	23,13	4,63	56,81	75,90	31,30	119,40	< 0,001
Silent thyroiditis adults	26	59,28	20,13	3,95	51,15	67,42	20,70	96,90	< 0,001
anaplastic Ca	3	95,00	13,25	7,65	62,08	127,92	87,35	110,30	1
follicular Ca	42	76,89	21,16	3,26	70,30	83,49	47,38	124,00	0,015
foll papillary Ca	11	68,85	18,57	5,60	56,37	81,33	46,84	96,77	0,176
giant cell papillary Ca	10	83,69	26,88	8,50	64,46	102,91	51,10	113,40	1
invasive Ca	8	74,80	18,28	6,46	59,51	90,08	60,68	103,10	1
microcarcinoma	4	68,10	9,54	4,77	52,91	83,28	59,83	76,36	1
papillary Ca	73	80,40	19,87	2,33	75,77	85,04	50,72	121,80	0,031
unclassified Ca	13	75,91	20,72	5,75	63,38	88,43	47,38	110,30	1
All	1401	84,20	22,50	0,60	83,02	85,38	20,70	187,20	

Correlation analyses for Se and TSH, Se and BMI and of Se and thyreoglobulin failed to demonstrate any significant association. In a similar fashion, the ample biochemical analysis of the ski jumper control group with 124 parameters revealed no associations for Se in relation to white and red blood counts, electrolytes, liver and kidney function tests, cortisol, testosterone, iron, folic acid, vitamin B12, immunoglobulins, and aminogram data including methionine levels.

## Discussion

### General evaluation in relation to external reference levels

Our study was designed as a controlled one in order to evaluate the levels of nutritional antioxidants, i.e. Se, Zn and vitamin C, in relation to morphological and functional parameters of benign and malignant thyroid disease. These nutritional parameters were chosen due to their physiological role in inflammation [46] as well as in anti-oxidative processes [47]. Oxidative processes in general are matter of current research in both benign and malignant disease since they are linked to aging, to general disease conditions as well as to increased mortality rates in the general population [27,30,48-51]. Through the use of local control groups we expected to be able to differentiate the normal and the pathological conditions [52]. Based on one way ANOVA analysis H_o _could be rejected for Se levels, i.e. Se levels are indeed different between patients and controls.

From a nutritional, qualitative point of view, our study documents diminished levels of vitamin C and Se in our patients as compared to recent data from the SU.VI.MAX study [34]. On the other hand, plasma Zn levels were found to be unrelated to thyroid function or morphology. Due to the predominantly intracellular distribution of Zn [53-59] a different analytical approach, i.e. determination of erythrocyte Zn levels, might have been more adequate to differentiate patients with hyperthyroidism from those with thyroiditis [60-64] as well as those with thyroid associated orbitopathy [46].

### The role of vitamin C in thyroid disease

Clinical studies on the importance of vitamin C in relation to thyroid disease are scarce. In 1977 Dubey et al. described low levels of ascorbic acid in patients with hyperthyroidism [65]. Similar results have been presented by Ademoglu et al. [66] and Alicigüzel et al. [67]. Mohar Kumar et al. described also low levels of vitamin C in hyperthyroidism, while at the same time lipid peroxides, glucose, and HbA1C levels were elevated [68]. It follows that both data from the literature as well as from our study show that vitamin C levels can be low in patients with benign thyroid disease. Low levels of vitamin C can predispose to endothelial changes [69,70], a situation which can also be negatively influenced by diminished thyroid function [71,72]. Our failure to demonstrate an association between vitamin C levels and benign thyroid disease could be explained through the relatively short half life of vitamin C of 10–20 days [73]. Nonetheless, interactions between vitamin C and Se have been described [26,74-77] by which low levels of vitamin C could affect Se metabolism and action.

### Se in relation to amino acids and other biochemical parameters and BMI

Early studies on the bioavailability of selenomethionine mentioned potential interactions between methionine and Se uptake [78]. Based on an extensive biochemical analysis with 124 parameters including an aminogram, we were not able to find any relation between Se and the parameters studied including methionine. The association between Se and methionine appears to be possible within the frame of malnutrition together with methionine deficiency as has been shown in experimental settings [79]. In our study, none of the patients presented malnutrition.

In the recent analysis of genetic SePP polymorphisms, Méplan et al. identified an association of BMI with Se levels for subjects having a BMI > 25 kg/m^2 ^[44]. In our study we were not able to find a similar association.

### Se in benign and malignant thyroid disease

Previous investigators have looked at Se levels in whole blood in relation to hyperthyroidism [13]. Beckett et al. reported lower values of Se for untreated Graves' disease (mean 88.9 μg/l) as compared to treated patients (mean 107.2 μg/l). The authors suggested that hyperthyroidism is responsible for low Se levels. Aihara et al. have also shown low erythrocyte levels of Se in cases of hyperthyroidism [80]. Using plasma determinations, Reglinski et al. have also shown low Se levels in hyperthyroidism [14]. These authors were able to document an increase in Se concentration after anti-thyroid treatment, however the mean Se value was still lower than that of the controls [14]. The question of Se normalization after remission of hyperthyroidism has been looked at recently by Wertenbruch et al. [81]. The authors could not find a normalization of Se in the remission phase: Se levels in Graves' disease without and with remission were 71.7 μg/l and 73 μg/l, respectively. In our study we have not found any relation between thyroid function parameters and Se levels in benign thyroid disease, so that we cannot lend support to Beckett's theory. Furthermore, we did not find any relation between Se and thyroid function parameters in the group of oncological patients who were taking T4 medication in a suppressive dose.

An analysis based on whole blood determinations of Se done by Kucharzewski et al. reported lower Se levels for thyroid carcinoma, nodular goiter, Graves' disease and thyroiditis as compared to normals [15]. While their study revealed lower Se levels in the 21 patients with thyroid carcinoma, there was no information as to the histological type of carcinoma. Out of 164 patients with thyroid carcinoma investigated by us only those with follicular or papillary types had low Se levels. Due to study design, we cannot fully confirm the observations obtained from the JANUS serum bank relating pre-diagnostic low Se levels with thyroid carcinoma [18]. Our data has discarded any association between Se and Tg.

### Selenium levels in pediatric patients

Dealing with Se levels in children differs from adult subjects since an age related increase can be seen. Se levels in children in the pre-school and school age are similar to those found in adults while younger patients have lower levels [82,83]. In 1992 Tiran et al. failed to observe this increase of Se levels for Austrian children [84,85]. The Se levels reported by Tiran et al. are far below the levels required for adequate expression of selenoproteins. Even though the mean level of Se in our pediatric patients is higher than that seen in 1992, a tendency to lower Se values can be seen in children with hyperthyroidism and with polyendocrinopathy. These situations have not been described before in the literature. Low levels of Se have been reported in Polish pediatric patients presenting thyroid diseases due to iodine deficiency [86]. Even after correction of iodine deficiency, children still had low Se levels [87]. Due to iodine supplementation programs in Austria, iodine deficiency is now rare [88], however Se levels can still be suboptimal, as we have documented here. On experimental grounds, Ruz et al. have nicely demonstrated the effects of single and combined deficiency situations involving iodine, Se, and Zn [20].

### Situations leading to low Se levels

Since our results point out the fact that a significant proportion of patients with thyroid disease have low levels of Se, it is important to analyze some of the known causes related to low Se levels [45,89,90] which might play a role in our patients. The central regulator of Se levels is the adequacy of supply, so that malnutrition in different degrees will lead to low Se levels [90-92]. Minor surgical interventions [93] and intensive care situations [94,95] can also induce a drop of Se concentrations. Nutritional studies have shown that feeding a low Se diet for 30 days can induce a drop of the plasma Se levels [96]. None of these conditions, however, were found in our subjects.

Stressful life events have been implicated in the etiology of thyroid disorders [97-103] and psychological stress per se can lead to diminished levels of Se [104]. In addition to this both physical and psychological stress can also induce changes in trace elements [105], e.g. to low levels of Mg [106], and low Mg levels can affect Se absorption [107,108]. In a previous study we have documented an altered relationship between Mg and Ca in patients with thyroid associated orbitopathy [46], thus lending support to this postulate.

In a general sense, viral infections can be considered to pose a stress to the organism [109-114]. Se deficit can increase the virulence of some infections such as with coxsackie virus in relation to heart disease [115]. Several authors have even proposed a viral etiology of thyroiditis, however this has not been confirmed neither in De Quervain's thyroiditis nor in Graves' disease [116-119]. Should a Se deficiency be present a-priori, viral infections can turn aggressive at least in animal models [120]. Similar associations have been described in HIV infections [121], however Beltran et al. could not find evidence for an autoimmune mechanism of thyroid disease in HIV patients [122].

Low Se levels can be seen in association with the acute phase of several pathological conditions [123]. Maehira et al. extended these observations by in-vitro experiments where it could be shown that low Se influences the activation of NF-Kappa-B, whereas a state of normal Se inhibits the NF-Kappa-B mechanisms that can lead to increased CRP levels [124]. An inflammatory reaction appears to depend on the interaction between IL-6, TNF and selenoprotein S [125]. Selenium and parameters of infection or inflammation have been recently described as a situation of negative correlation between Se and CRP and IL-6 [95]. Results from the MONICA/KORA Augsburg study have shown that normal Se levels are related to vitamins C and E as well as to low CRP values [126].

In a study presented by Pearce et al. [127] a central role of elevated CRP levels in thyroid disease could not be found. Severe health impairment such as found in sepsis and multiorgan failure, on the other hand, can indeed alter Se levels [95]. Clinical practice with thyroid disease patients however, does not lend evidence to the presence of a severe infection at the time an out-patient consultation takes place. A different situation could be expected in cases of chronic infection or parenteral nutrition [128].

### Biochemical changes in Se deficiency

Besides the diminished levels of Se in cases of deficiency a series of Se-dependent proteins can be found to be altered in such situations. This has been shown elegantly in Finland after Se supplementation was introduced in the 1980s: supplementation with Se led to an increase of the levels of Se and of SePP. Once Se repletion was achieved, SePP levels did not change significantly when Se was given [42]. In cases of Se deficiency, the changes in SePP are tissue specific [129]. In addition to this feature a recent study has documented the influence of gender and genetic polymorphisms on the response pattern of SePP to Se supplementation [44]. Analyses of Se together with selenoproteins, which can be considered to be a better indicator of Se status [130,131], have not been carried out in cases of thyroid disease.

### What to do in face of low Se levels in patient with thyroid disease?

The description of deficient nutritional situations belongs to the classical approach used in nutrition research in order to identify causes and to treat them by substitution [132]. In the previous section we have tried to delineate some of the changes that can be expected to happen within the setting of low Se levels in relation to inflammation and which might change during supplementation. The use of nutrient supplementation which is not based on the determination of nutrient levels cannot be expected to achieve beneficial effects since the needed dose and the target parameters might be incorrect. In addition, the chemical form of the nutrient has to be chosen adequately [133]. Reading the description of the individual studies cited in the meta analysis done by Bjelakovic et al. [134] it is easy to recognize why the analysts did not find positive effects of supplementation procedures: some studies did not measure the basal levels prior to intervention, and the doses and chemical forms of the preparations differed greatly.

At the time we carried out this study, several Se supplementation trials for patients with autoimmune thyroid disease were published [135-138]. In these studies the common denominator was the drop in the level of thyroid antibodies, i.e. a better nutritional status induced changes in the humoral arm of the immune system. The study by Gärtner et al. utilized 200 μg of a liquid formulation of sodium selenite over a period of 3 months. In the verum group Se levels increased from 0.87 to 1.09 μM; at the same time thyroid peroxidase antibody levels dropped by 37% [135]. Duntas et al. [136] carried out "arbitrary" evaluations in a subgroup of the patients studied. They found a basal Se level of 75 μg/l. Intervention was done using selenomethionine at a dose of 200 μg/d and were able to observe a greater drop of thyroid antibody levels of 46% after 3 months, and of 55.5% after 6 months of therapy. Turker and colleagues compared the efficacy of 100 μg and 200 μg of selenomethionine concluding that better effects could be reached by the higher dose [137]. Mazopakis et al. [138] used a dose of 200 μg selenomethionine and observed also a drop in the levels of thyroid peroxidase antibodies. None of the studies found changes in the levels of thyroid hormones. Finally Negro et al. [139] studied a group of pregnant women who presented thyroid peroxidase antibodies. The base-line Se levels ranged between 78.2 and 80.9 μg/l. Under treatment with 200 μg selenomethionine, Se levels remained significantly elevated, app. 105 μg/l, while the untreated group showed a transient drop of Se at 30 weeks of pregnancy. Se supplementation was associated with a lower incidence of hypothyroidism and thyroid inflammation. In our previous preliminary series Se substitution with 200 μg of selenomethionine was instituted when Se levels were lower than 70 μg/l. Besides a drop of thyroid antibodies, we were also able to document an increase of thyroid function, i.e. latent hypothyroidism was corrected [140].

## Conclusion

From a global point of view, we can state that nutritional deficiencies in relation to Se and vitamin C have been found in our study of Austrian patients. These findings contradict the official Austrian opinion contained in the Austrian Nutrition Report 2003 [141].

Within the wide spectrum of patients with thyroid disease studied herein, the most salient feature is that of diminished levels of Se in cases of sub-acute and silent thyroiditis, as well as in the setting of pediatric patients with hyperthyroidism and polyendocrinopathy, and in cases of differentiated thyroid carcinoma. The mechanisms that lead to low Se levels are not yet known. Loss of differentiation of thyroid carcinoma has been recently postulated to be related to Se metabolism and indirectly to octreotide uptake [142].

## Competing interests

The author(s) declare that they have no competing interests.

## Authors' contributions

Thyroid disease patients were examined by RM, FK, MO, and AK. RM, MO, and FK managed the data base for the patients with benign thyroid disease. MS worked with the oncological thyroid cases. HT carried out the laboratory determinations of the nutritional factors. KK examined and managed the pediatric patients. RM and HM examined and managed the clinical and laboratory data of the control population for Se (WOMED group). RM and HM wrote the manuscript. All authors read and approved the final manuscript.

## Pre-publication history

The pre-publication history for this paper can be accessed here:


